# Global Forum 2015 dialogue on “From evidence to policy – thinking outside the box”: perspectives to improve evidence uptake and good practices in the African Region

**DOI:** 10.1186/s12913-016-1453-z

**Published:** 2016-07-18

**Authors:** Joses Muthuri Kirigia, Charles Ok Pannenborg, Luis Gabriel Cuervo Amore, Hassen Ghannem, Carel IJsselmuiden, Juliet Nabyonga-Orem

**Affiliations:** 1 Health Systems and Services Cluster, World Health Organization, Regional Office for Africa, B. P. 06, Brazzaville, Congo; 2 The World Bank, Washington, D.C, USA; 3 Research Promotion and Development, Pan American Health Organization/World Health Organization (PAHO/WHO), Washington, D.C, USA; 4 Department of Epidemiology, University Hospital Farhat Hached, Sousse, Tunis Tunisia; 5 Council on Health Research for Development (COHRED), Geneva, Switzerland

**Keywords:** National health research systems, Knowledge translation, Support of research, Diffusion of innovation, MDGs and SDGs

## Abstract

**Background:**

The Global Forum 2015 panel session dialogue entitled “From evidence to policy – thinking outside the box” was held on 26 August 2015 in the Philippines to debate why evidence was not fully translated into policy and practice and what could be done to increase its uptake. This paper reports the reasons and possible actions for increasing the uptake of evidence, and highlights the actions partners could take to increase the use of evidence in the African Region.

**Discussion:**

The Global Forum 2015 debate attributed African Region’s low uptake of evidence to the big gap in incentives and interests between research for health researchers and public health policy-makers; limited appreciation on the side of researchers that public health decisions are based on multiple and complex considerations; perception among users that research evidence is not relevant to local contexts; absence of knowledge translation platforms; sub-optimal collaboration and engagement between industry and research institutions; lack of involvement of civil society organizations; lack of engagement of communities in the research process; failure to engage the media; limited awareness and debate in national and local parliaments on the importance of investing in research and innovation; and dearth of research and innovation parks in the African Region.

**Conclusion:**

The actions needed in the Region to increase the uptake of evidence in policy and practice include strengthening NHRS governance; bridging the motivation gap between researchers and health policy-makers; restoring trust between researchers and decision-makers; ensuring close and continuous intellectual intercourse among researchers, ministry of health policy-makers and technocrats during the life course of research projects or programmes; proactive collaboration between academia and industry; regular briefings of civil society, media, relevant parliamentary committees and development partners; development of vibrant knowledge translation platforms; development of action plans for implementing research recommendations, preferably in the context of the Sustainable Development Goals; and encouragement of competition on NHRS strengthening and research output and uptake among the countries using a barometer or scorecard to review their performance at various regional ministerial forums and taking into account the lessons learned from the MDG period.

## Background

The purposes of this paper are to summarise the progress made towards the achievement of the health-related Millennium Development Goals (MDGs) and in strengthening the national health research systems’ (NHRS) governance in the World Health Organization (WHO) African Region; debate the reasons for the low uptake of evidence in public health policy and practice and the possible actions for increasing its uptake; and highlight what partners could do to increase the use of evidence in the Region.

Majority of the countries in the Region had not achieved most of the health and health-related MDGs 1, 4, 5, 6 and 7 by the end of 2015. Of the 47 countries in the Region, 31 had not achieved target 4A on reducing child mortality, 41 had not attained target 5A on reducing maternal mortality, 35 failed to achieve target 6B on reducing malaria incidence, 18 had not met target 6C on reducing tuberculosis incidence, and 3 had not achieved target 6A on reducing HIV incidence [1]. Similarly, most of the countries did not attain the MDGs related to the broader determinants of health. For instance, 30 countries had not achieved the target of reducing the number of underweight children aged less than 5 years, and MDG 7’s targets of increasing the coverage of water provision and sanitation had not been achieved by 24 countries and 39 countries, respectively. The inability to achieve the health and health-related MDGs has been attributed to the persisting weakness of the national and sub-national health systems, the systems that address the social determinants of health, and the national health research systems (NHRS) [1].

Even before concluding the health MDG agenda, the countries are forced to brace themselves to transition to the post-2015 Sustainable Development Goals’ (SDGs) agenda [2], whose SDG 3 is on ensuring healthy lives and promoting the wellbeing of all at all ages, and target 3.8 is on achieving universal health coverage (UHC), including providing financial risk protection and access to quality essential health care services and to safe, effective, quality and affordable essential medicines and vaccines for all. Movement towards UHC entails simultaneous progress in the expansion of the range of health and health-related services available, the covered proportion of the cost of those services, and the proportion of the population covered [3, 4].

The *World health report 2013* [5] illustrates with 12 case studies that research illuminates the path to UHC and to better health. Nine of the case studies are on the diversity of the problems tackled using randomised controlled trials: insecticide-treated mosquito nets to reduce childhood mortality in 22 sub-Saharan African countries; antiretroviral therapy to prevent sexual transmission of HIV in Africa, Asia, Latin America and North America; zinc supplementation to reduce pneumonia and diarrhoea in young children in Bangladesh; telemedicine to improve the quality of paediatric care in Somalia; new diagnostics (Xpert MTB/RIF) for tuberculosis in Azerbaijan, India, Peru and South Africa; the “polypill” to reduce deaths from cardiovascular disease in India; combination treatment with sodium stibogluconate (SSG) and paromomycin compared to SSG monotherapy for visceral leishmaniasis in Ethiopia, Kenya, Sudan and Uganda; task shifting in the scaling up of interventions to improve child survival in Bangladesh, Brazil, Tanzania and Uganda; and insurance in the provision of accessible and affordable health services in Mexico. The other three case studies are on operational research that led to improved access to emergency obstetrics care in rural Burundi, conditional cash transfers to improve the use of health services and health outcomes in low and middle income countries, and affordable health care in ageing populations in the Czech Republic, Germany, Hungary, the Netherlands and Slovenia [5].

SDG 3 is much more ambitious than the outgoing health MDGs. The process of planning and developing country road maps for scaling up health and health-related policy and programmatic interventions for UHC requires generation and utilisation of contextualised evidence. Examples of the evidence needed include information on the burden of disease; baseline population coverage of health and health-related interventions, including household survey and health behaviour data; effectiveness and cost-effectiveness of interventions and delivery models; packages of essential health services to be accessed by everyone in need; economic feasibility of prepaid health financing mechanisms; and sociocultural, political and professional feasibility of the services [5]. This kind of evidence will not be forthcoming or available where NHRS are not functional [6]. That is why research, innovation and domestic technology development are prominent targets of SDG 9, which is on building resilient infrastructure, promoting inclusive and sustainable industrialisation and fostering innovation.

To stand a fair chance of attaining SDG 3, African Region countries need to have functional NHRS that facilitate the production and use of pertinent evidence in public health policy and practice. Between 2000 and 2014, the Region accounted for only 1.3 % of the global output of scientific publications [7], which is not commensurate with its 12 % share of the global population. The low research output is partially attributable to the weak NHRS governance. For example, of the 47 countries in the Region, 24 have not defined their health research policy at all, 28 have no laws or regulations governing research, 25 have not developed a strategic research plan and 22 lack a national priority agenda on research for health [8]. The low research output partially accounts for the dearth of development and innovation of health restoring or sustaining products [9]. Weaknesses in NHRS are exacerbated by the significant challenges in training and retaining of academic, scientific or research human resources for research for health and biomedical purposes.

## Discussion

### Forum 2015 session dialogue method

The Global Forum on Research and Innovation for Health 2015 took place in Manila, Philippines, 24–27 August 2015. It was co-organised by the Council on Health Research for Development (COHRED), the Philippine Department of Science and Technology, the Philippine Department of Health and the Philippine Council for Health Research and Development. Its aim was to identify research and innovation solutions to the world’s unmet health needs. The programme had two pillars focusing on increasing the effectiveness of research and innovation for health and on the role of research and innovation. The proceedings were organised under six broad themes: social accountability for research and innovation for health, increasing investment for research and innovation for health, country-driven capacity building for research and innovation for health, research and innovation for food and nutrition safety and security, research and innovation to safeguard health in megacities and research and innovation in disaster preparedness.

This paper reports on the 75-minute debate that took place on 26 August 2015 in the session entitled “From evidence to policy – thinking outside the box”. The debate focused on how to improve the uptake and use of research-derived evidence in creating better health policies and practices and how to get health in all policies. The session started with introductions of the five keynote speakers by the moderator, who was from the World Bank. The speakers were from the University Hospital Farhat Hached in Tunisia, Institute of Global Health Innovation of the Imperial College London, WHO Regional Office for Africa, WHO Regional Office for Europe, and Pan American Health Organization/WHO Regional Office for the Americas. The speakers took an average of six minutes. The floor was then opened for questions and comments from the audience. The speakers noted the questions and comments, and subsequently responded to them. The moderator provided a summary of emerging recommendations at the end of the session.

The session was attended by high level representatives from governments including policy-makers and senior technical staff from the ministries of education, science and technology, environment and health; civil society organisations (CSOs); international organisations; private for-profit sector; private not-for-profit sector; academic and research institutions, including faculty members and students; and academic journal editors, including the Asia Pacific Association of Medical Editors, Nature Partner Journals and British Medical Journal [BMJ] Open. Approximately 300 people participated in the session.

A conscious effort has been made by the authors to buttress the recommendations from the debate with pertinent published literature. The main limitation of the paper is that the verbatim proceedings were not audio recorded, so what is reported here is a summary of the notes taken by the panellists.

### Forum 2015 session debate issues

#### Why the low the uptake of evidence in public health policy and practice?

The Global Forum 2015 debate attributed African Region’s low uptake of evidence in public health policy and practice to a number of factors.

First, there is a big gap in incentives and interests between research for health researchers and public health policy-makers. Researchers and policy-makers are known to pursue different interests, which in itself presents a discord in the uptake of research evidence. Choi et al. [10] argue that the discord is premised on differences in mentalities and imperatives of scientists and policy-makers, among which are pursuance of different goals and career paths, perception of time, lack of mutual trust and respect, and different attitudes towards research [10]. While researchers attach a lot of importance to scientific rigour, policy-makers look for important, timely information from informal evidence-generation mechanisms like real experiences that can drive decision-making, considering the political and contextual realities [10]. Even with the best of intentions, because of the nature of the system, researchers end up being driven by the need to publish to retain their scientific or research jobs and also to merit academic promotion (the so-called publish or perish notion); are inclined to publish in high impact journals for prestige among their peers; feed their research into teaching and learning; work to gain goodwill with research funders [11, 12]; and earn royalties on intellectual properties [13, 14]. This is limiting in a number of ways. Academic journals may not be the best way to disseminate evidence in low income countries, where access to journals is limited owing to Internet connectivity handicaps and cost, the reading culture is weak, literacy levels are relatively low, and the technical or scientific language used may not be easily accessible to policy-makers [15].

Policy-makers on their part are forced to do a balancing act in striving to manage enormous – and at times catastrophic – vested interests and pressure from politicians; donors; professional trade unions; civil society lobby groups; the community, whose sense has been heightened by the emergence of patient charters; and the ministry of health’s priority programmes such as those on HIV/AIDS, tuberculosis, malaria, maternal and child health, as well as the ministry of finance’s budget pressures and limitations. Added to these are the specific manager’s whims, personal preferences and decision-making style, which might be influenced by the emotions of the moment or day [16–19]. Health policy-makers are always trying to balance scarce resources against unlimited clinical and public health needs and demands [20–22], and they need to make timely decisions that need to consider many contextual issues [19].

Second, there is limited appreciation on the side of researchers that public health decisions are based on multiple and complex considerations, for example those related to circumstances of political, economic, social, cultural, and windows-of-opportunity nature, among which evidence may or may not necessarily occupy a prominent place [19, 23, 24]. For example, Hennink and colleagues [25] caution against research recommendations that ignore affordability concerns, while Humphreys and Piot [26] caution against recommendations that are contrary to social norms and recommend weighing the possible electoral consequences of the different policy options.

Third, there is often the perception among intended research users, especially policy-makers, that research evidence is not relevant to local contexts. The perceived quality and relevance of the research evidence [27, 28], the credibility of the researchers [29, 30] and the extent to which evidence resonates with social and cultural norms and political interests also do impact its uptake in decision-making [31, 32]. There are instances where high quality evidence has not been taken up in policy due to the lack of political support, like the case of medical male circumcision in Uganda [33] or indoor residual spraying for malaria vector control in Zimbabwe [34]. An additional consideration has been the different time horizons that underpin scientific research and policy-making. The decision-makers at times use readily available, unpublished literature and information from newspapers to take decisions, whereas scientific research takes time to design, conduct, write, peer review, publish, and properly summarise and package to be of use to policy-makers [35].

Fourth, the absence of institutionalised and systematic knowledge translation platforms in the majority of African countries reduces the opportunities for dialogue between researchers and policy-makers, which contributes to the widening of the communication and trust gaps between the key constituencies and stakeholders. By the end of 2014, 15 of the 47 countries in the African Region had a knowledge translation platform to facilitate the uptake of evidence in policy development and practice [8]. This represented substantial improvement in the last decade, but progress remains insufficient. Institutionalised and systematic knowledge translation platforms have been deemed helpful where they exist and are functional, for example in Uganda, where the malaria treatment policy was changed following the generation of evidence from drug efficacy studies [29], and in Kenya, Malawi and Nigeria, where there was uptake of evidence from operational research [36].

Fifth, there has been sub-optimal collaboration and engagement between industry and research institutions, including universities and other tertiary institutions across Africa, which negatively affects the quality and relevance of learning and research and reduces the chances of translating research findings into product innovation and development. This likely accounts for the fact that out of approximately 850 new therapeutic products (drugs and vaccines) registered worldwide in 2000–2011, only 37 were indicated for common and serious diseases such as malaria, tuberculosis, diarrhoeal diseases, neglected tropical diseases and other diseases of poverty that disproportionately affect Africa [9].

Sixth, the lack of involvement of CSOs in research, development and innovation contributes to the low uptake of evidence. From the interactions at the Global Forum 2015, it was clear that progressive involvement of CSOs in research and development within functional NHRS has catalysed the uptake of evidence in policy and practice in Asia, Latin America, Europe and North America. In those continents, CSOs continue to play an important advocacy role by demanding that public decisions be based primarily or as much as possible on science and other relevant evidence. CSO advocacy and lobbying capacities have not been optimally tapped to further the uptake of evidence in the Africa Region and in many cases have been or are being politically suppressed. Doyle and Patel [37] highlight the inadequate capacity of CSOs to navigate the political terrain and to influence policy. Furthermore, the specific challenges faced by CSOs in low income countries need to be addressed, among which are weak internal organisations, lack of independence and varied capacity.

Seventh, the lack of structures to enable meaningful engagement of communities in the research process has been highlighted as a major bottleneck to the uptake of evidence [38, 39]. It was also clear during the Global Forum 2015 proceedings that in the rest of the world deliberate efforts are being made to increase societal/community awareness of the role of research and innovation in human development. In many countries, communities, including patients, are increasingly viewed as active partners in research. In the best of cases, a culture of critical enquiry and investigation is encouraged and nurtured from childhood to old age and from kindergarten to postgraduate levels. In the Africa Region community awareness of and involvement in research for health are limited, and the research and innovation culture is growing only slowly, as seen in national research, science and technology symposia and competitions. There is need for consciously planned efforts to cultivate research and innovation awareness among the community and more importantly to mainstream research and innovation in student and teacher or lecturer training curricula at all levels of the national education system.

Eighth, the Global Forum 2015 participants opined that the absence of an official regional policy and strategy on research for health may hamper development of NHRS, including the uptake of evidence. Having a regional policy and strategy on research for health for Africa [40] and accurate indicators of progress within the structure and processes of NHRS will likely result in actionable research and generation of momentum in advancing such systems in the countries, as has happened in other regions [41, 42].

Ninth, researchers in the African Region often fail to deliberately engage the media over the life course of research projects to create public awareness and to communicate research findings and advocate for their use in decision-making. The media have been shown to be useful in community mobilisation and raising their awareness of evidence, thereby empowering them to demand that certain proven, cost-effective polices be implemented [43]. The experiences from different regions shared at the Global Forum 2015 indicated that active engagement of the media in the life course of research for health projects has helped to bolster the translation of evidence into policy, practice and innovation.

Tenth, there seems to be limited awareness and debate in national and local parliaments across the African Region on the importance of investing in research and innovation. This is reflected by the fact that 22 of the 47 countries in the Region do not have a budget line for research for health [8]. So far only two countries have complied with the recommendation of the Commission on Health Research and Development and the Algiers Ministerial Declaration on Research for Health to allocate at least 2 % of the national health sector budget to health research [44]. The Global Forum 2015 participants argued that the limited awareness of parliamentarians of the role of research in health and their little engagement in it might explain the paucity of research and innovation champions among the countries of the Africa Region.

Lastly, the low uptake of evidence was attributed also to the dearth of research and innovation (science) parks in the African Region. It was clear at the forum that some Asian and Latin American countries, having drawn lessons from the industrialised countries of the Organisation for Economic Co-operation and Development, were investing heavily in the development of research and innovation parks. Such parks act as magnets for talent and incubation centres for ideas, some of which find their way into product development and innovation. In the African Region, only South Africa and Uganda reported that they were developing such structures.

#### What can African countries do to increase the uptake of evidence?

The Global Forum 2015 debate highlighted a number of actions that were needed to increase the uptake of evidence in policy and practice.

First, strengthen NHRS governance to create an enabling environment for research and innovation. Every country should ensure that it has a national health research policy, a strategic plan, an agenda for priority research for health and pertinent legislation.

Second, bridge the gap between researchers and health policy-makers. It is important for researchers to realise and acknowledge that they are partly responsible for the problem of low evidence uptake. To provide incentives for researchers to conduct research that serves policy, practice (innovation) and/or product development purposes, it may be beneficial for a national research community, together with their policy-makers, to devise and implement a system of weighting the impact of research articles in terms of their uptake in policy, practice and product development. For example, an article whose findings influence public health policy, practice and product development could be given a score of 1 or 100 %, one that influences policy could be given 0.75 or 75 %, one impacting only public health or clinical practice could be given 0.5 or 50 %, and one with no impact on policy, practice or product development could receive 0.25 or 25 %. If developed in a participatory manner and adopted, such weighting might contribute to the modification of the incentive structures and behaviour of researchers to favour research orientations that more effectively address public health challenges confronting the Africa Region. Another consideration would be the development of a prioritised national research agenda in a participatory manner to ensure that researchers’ and policy-makers’ interests are aligned.

Third, build trust between researchers and decision-makers of the kind that was characteristic of such relationships in most countries in the African Region around the period of political independence [45, 46]. This will require changing the firmly entrenched negative perceptions and, indeed, the mind-set. Some countries have used memorandums of understanding between research for health institutions and the ministry of health to build bridges. The memorandums often cover research, monitoring and evaluation of research, and short-term (on-the-job) and long-term human resource capacity strengthening. Institutionally, the establishment of national academies of science can equally be instrumental in building bridges of trust, by including as their leading members scientists and intellectual leaders and recognised policy-makers, politicians and other national leaders. Such joint forums would subsequently need to be given public prominence.

Fourth, ensure close intellectual intercourse between researchers and the ministry of health’s decision-makers and technocrats during the entire research projects’ life course. The panellists noted that from their experience, in situations where that constant engagement exists, the research recommendations invariably easily find their way into policy and practice. However, such close engagement requires investment in money and time, which ought to be foreseen and factored into the research projects’ cost.

Fifth, introduce regular briefing of CSOs and the media during a project’s lifecycle, which may increase the uptake of its research findings and recommendations. We hasten to add, however, that written media briefings have proved to be more effective than verbal briefings, as in some cases these stakeholders have been found to misrepresent the facts [15].

Sixth, grow and nurture collaboration between academia (and research institutions) and industry to increase research uptake in innovation and product development. Lessons from economically developed and emerging economies indicate that close collaboration between academia and industry enhances the quality and relevance of teaching, funding for research, overall sustainability of academic institutions, and volume of research and innovation outputs, and increases the rate of translation of such outputs into product development. At the Global Forum 2015 the example of Singapore’s research and development and innovation policies and practice over the last 25 years was highlighted in this respect. Also, including the most respected research leaders from industry in the national academy of sciences has been shown to be an important long-term capacity-building element in creating a viable research-academia-industry constituency in a country.

Seventh, researchers in the applied sciences ought to avoid making research for health recommendations that are generic, but instead should ensure that their recommendations are feasible, implementable and scalable to the furthest extent possible. In addition, they should work with those expected to utilise the evidence to develop action plans that contain SMART (specific, measurable, attainable, relevant and time-bound) actions, cost estimates, possible sources of funding and names and roles of persons responsible for their execution.

Eighth, establish inclusive knowledge translation platforms such as the Evidence Informed Policy Network (EVIPNet) [47]. Such platforms ought to be made up of multidisciplinary and multi-stakeholder teams, drawing experts from all the relevant disciplines. In addition, they should include policy-makers, selected technocrats from the ministry of health and representatives of civil society, the media and, to the utmost extent possible, the national parliament. The role of the platform is to provide inputs to inform policy options and to create the environment where these can be discussed with the participation of relevant stakeholders and where policy-makers can assess the potential benefits and risks for each policy option and the feasibility of its implementation, considering the contextual issues. Examples of the tools used for this assessment are policy briefs, research syntheses, plans of action for implementing recommendations/findings, advocacy for uptake of evidence in decision-making, deliberative dialogues, rapid response mechanisms, and technical advice for policy-makers and implementers of interventions [48].

Ninth, make health research and innovation a part of the societies’/communities’ mantra to empower them to demand that health development decisions be informed by research evidence.

#### What can partners do to help African countries increase the uptake of evidence?

The Global Forum 2015 debates highlighted the actions that the relevant international partners ought to undertake to support the implementation of the research-related recommendations from the various high level ministerial forums held in Africa.

First, provide technical support for building/strengthening NHRS. For example, COHRED has developed three tools that would help African countries to improve governance of research. These are (1) a cloud-based research ethics review software called RHInnO Ethics, which is already being used in 8 African countries and 27 research ethics committees [49]; (2) the Fair Research Contracting service, which provides legal expertise using guides, the web and online/telephone or on-site consulting services and course work learning opportunities for research administrators [50]; and (3) the COHRED Fairness Index, which is a global certification mechanism for safeguarding fairness in research contracting [51, 52]. WHO also provides technical support to Member States for developing national health research policies, strategic plans, research agenda, and standards and operational guidelines for ethics review of health-related research [53, 54], as well as for training of national research ethics committees [55] and for implementation of research [56].

Second, support countries to develop human resources for health research. For example, the Special Programme for Research and Training in Tropical Diseases (TDR) has been providing training grants for doctor of philosophy, master’s and fellowship training in product development; customised workshops; mentorship and networking opportunities; and research support to strengthen capacity in low and middle income countries [57].

Third, support the countries to establish and maintain knowledge translation and sharing platforms such as EVIPNet to facilitate the use of research in health decision-making.

Fourth, global health initiatives and development agencies, including the United Nations agencies, ought to devote at least 5 % of their overall health investment portfolio to support the strengthening/building of NHRS [44, 58]. This was a recommendation originally made by the 1990 Commission on Health Research and Development [58] and reiterated in the Algiers Ministerial Declaration on Research for Health [44].

Fifth, it is a fact that, rightly or wrongly, many global health development partners have significant influence on African countries’ processes for developing health policies and plans. This is attributed to the critical reliance of a substantial proportion of African countries on donor funding for health development, especially for investment budgets (as opposed to mostly domestically financed recurrent budgets), and this does not necessarily strengthen governance and stewardship by governments [59]. There is evidence that involving global partners in the life course of research projects can be helpful in advocacy for and uptake of findings and recommendations in national or local health policies and practice [60].

Sixth, provide funding for research on priority problems identified by the countries and contained in their national health research agenda or identified at the regional high level ministerial meetings. For instance, both the Abuja and Accra communiques from the High Level Ministerial Meetings on Health Research in Africa identified as research priorities (1) health systems research, including health policy and human resources research; (2) infectious diseases such as malaria, tuberculosis, HIV/AIDS, emerging infections, and neglected tropical diseases such as African trypanosomiasis, Buruli ulcer, leishmaniasis and lymphatic filariasis, that require improvement in prevention, diagnosis, treatment, control and surveillance; (3) sexual and reproductive health; (4) newborn and child health; (5) non-communicable diseases, including cardiovascular disease, diabetes, cancer, sickle cell disease, injuries and occupational diseases; (6) malnutrition (undernutrition and obesity); and (7) mental health, including drug and substance abuse [61, 62]. TDR is an example of organisations that support (1) the development of new tools and implementation research for infectious diseases of poverty; (2) finding ways of helping vulnerable populations to develop resilience and survive climate change; (3) elimination of malaria, visceral leishmaniasis and onchocerciasis; and (4) training of community volunteers in remote or low resource settings to diagnose and treat childhood fever-related illnesses like pneumonia, diarrhoea and malaria [57].

Seventh, support regional coordination and South–South cooperation and networking in research, development and innovation for health [62].

Eighth, support the full implementation of the regional strategy on research for health that was adopted by the ministers of health from all the 47 WHO African Member States during the Sixty-fifth WHO Regional Committee for Africa session [40].

Ninth, encourage competition among countries in NHRS strengthening and research output and uptake through the use of regional and sub-regional scorecards [63]. Reviews of these can be conducted, discussed and subsequently published at regional, annual or periodic meetings of such overarching agencies as the African Academy of Sciences [64], the Association of African Universities [65], the Inter-University Council of East Africa [66], etc. In addition, the NHRS performance scorecards or league tables could potentially be discussed at the African Union, regional economic communities and WHO Regional Committee forums to further stimulate debate and healthy competition.

## Conclusions

A majority of African Region countries will not achieve the health and health-related MDGs owing to weaknesses in their national and local health systems, systems that address the social determinants of health, and NHRS. The weak NHRS across Africa account for the low research for health output and its uptake in public health policy and practice. The debate at the Global Forum 2015 attributed the low uptake of evidence partly to weak NHRS governance; the incentive gap between researchers and policy-makers; a deficit in trust, confidence and respect between researchers and policy-makers [67]; a lack of appreciation on the side of researchers that public health decisions are a function of many factors; the absence of knowledge translation platforms in most countries; and sub-optimal collaboration and engagement between researchers and research institutions on the one hand and industry, communities, CSOs, the media and parliamentarians on the other hand.

Some of the actions suggested during the debates for increasing the uptake of evidence in policy and practice include (1) strengthening NHRS governance; (2) bridging the gap in motivation between researchers and health policy-makers; (3) restoring trust between researchers and decision-makers; (4) ensuring close intellectual intercourse between researchers and ministry of health policy-makers and technocrats during the entire life course of a research project or programme; (5) fostering collaboration between academia and industry; (6) regularly briefing civil society, the media, relevant parliamentary committees and development partners; (7) developing vibrant knowledge translation platforms; (8) developing action plans for implementing research recommendations; and (9) encouraging competition on NHRS strengthening and research output and uptake among countries using a barometer or scorecard. Peer review of the scorecard could take place at various regional economic community forums, the African Union and the Regional Committee’s ministers of health sessions, as well as at regional academic, scientific and research meetings.

These actions will not happen in a vacuum. The global Strategy on Research for Health approved in 2010 by the World Health Assembly already is giving direction and is relevant to some of the existing platforms and efforts that have led to progress in Africa. For example, the development of the EVIPNet platform, the Regional East African Community Health Policy Initiative [68] and the International Clinical Trials Registry Platform and the subsequent Pan African Clinical Trial Registry that feeds into it are all linked to the WHO strategy, and resulted from the deliberations held in the WHO African Advisory Committee on Research for Health. WHO work also builds upon the huge and commendable contributions made by COHRED over more than two decades in the areas of national health research systems, research for health and ethical review systems [69]. The adoption of the African Region Strategy on Research for Health by the Sixty-fifth Session of the WHO Regional Committee for Africa in November 2015 was an opportunity to regain momentum and seek further adoption of key strategies that contribute to better production and use of research for health.

## Abbreviations

COHRED, Council on Health Research for Development; CSO, civil society organisation; EVIPNet, Evidence-informed Policy Network; MDG, Millennium Development Goal; NHRS, national health research systems; SDG, Sustainable Development Goal; TDR, Special Programme for Research and Training in Tropical Diseases; UHC, universal health coverage; WHO, World Health Organization
